# Investigation of *Kluyveromyces marxianus* as a novel host for large‐scale production of porcine parvovirus virus‐like particles

**DOI:** 10.1186/s12934-021-01514-5

**Published:** 2021-01-25

**Authors:** Deqiang Yang, Lei Chen, Jinkun Duan, Yao Yu, Jungang Zhou, Hong Lu

**Affiliations:** 1 State Key Laboratory of Genetic Engineering, School of Life Sciences, Fudan University, 2005 Songhu Road, Shanghai, 200438 People’s Republic of China; 2 Shanghai Engineering Research Center of Industrial Microorganisms, 2005 Songhu Road, Shanghai, 200438 People’s Republic of China; 3 Shanghai Collaborative Innovation Center for Biomanufacturing (SCICB), East China University of Science and Technology, 130 Meilong Road, Shanghai, 200237 People’s Republic of China

**Keywords:** *Kluyveromyces marxianus*, Porcine parvovirus, Virus‐like particles, Vaccine, Immunity

## Abstract

**Background:**

Porcine Parvovirus (PPV) is a *Parvovirinae* virus that can cause embryonic and fetal loss and death and mummification in affected fetal pigs. Unlike conventional vaccines, virus-like particles (VLPs) inherit the natural structure of their authentic virions and highly immunostimulatory that can induce strong humoral immune and T cell responses with no risk of pathogenicity. The production of PPV VLPs is still a challenge based on traditional expression platforms due to their low yields and high culture costs. *Kluyveromyces marxianus* is a safe and fast-growing eukaryote that can get high biomass with low-cost cultures. In this study, we investigated the expression and downstream processes of PPV VLPs in *K. marxianus*, and the potential for effective stand-alone vaccines.

**Results:**

After optimization according to the codon bias of *K. marxianus*, the VP2 protein from Kresse strain was highly expressed. In a 5 L fermentator, the yield of PPV VLPs reached 2.5 g/L, quantified by HPLC, using a defined mineral medium after 48 h fermentation. Two strategies were established to purify intracellular PPV VLPs: (i) Using the cation exchange chromatography coupled with Sephacryl® S-500 HR chromatography to purify VLPs from the supernatants of pH adjusted cell lysates. (ii) Using anion exchange chromatography followed by cross-flow diafiltration to recover the VLPs precipitated in pH adjusted cell lysates. The purity of PPV VLPs reached about 95%, and total recovery was more than 60%. Vaccination of mice with the purified PPV VLPs induced high titers of specific IgG antibodies in sera, and showed hemagglutination inhibitions on both swine and guinea pig erythrocytes. Spleen lymphocyte proliferation and cytokines detection suggested the PPV VLPs produced by *K. marxianus* provoked the cellular immune and humoral immunity responses in mice.

**Conclusions:**

This is the highest production of recombinant PPV VLPs achieved to date. The superiorities, Generally Recognized As Safe (GRAS), high production, short lead time, and low cost, make *K. marxianus* a greatly competitive platform for bioproduction of PPV VLPs vaccine.

## Background

Parvoviruses are small, non-enveloped DNA viruses belonging to the *Parvoviridae* taxonomic family and are divided into two subfamilies, the *Parvovirinae* and the *Densovirinae*, that infects vertebrates and arthropods respectively [1]. In the *Parvovirinae* subfamily, parvoviruses in the genera *Protoparvovirus*, *Bocaparvovirus*, *Copiparvovirus* and *Teraparovirus*, were also designated as porcine parvovirus types 1 through 6 [2]. Classic porcine parvovirus (porcine parvovirus type 1, PPV1), newly named *Ungulate protoparvovirus* 1, was first isolated from a contaminant pig kidney culture in 1965 in Germany [3]. In the past two decades, with the development of molecular biology techniques, six new serotypes of parvovirus have been clinically discovered in pigs, and designated as PPV2 to PPV7 [1]. The PPV7 was first discovered in the USA in 2016 as a new serotype of PPV in the rectal swabs of healthy adult pigs by the metagenomic sequencing technology [4]. PPV was a major causative agent of the swine reproductive failures traditionally summarized as the stillbirth, mummification, embryonic death, and infertility (SMEDI) syndrome [5]. The incidence and severity of symptoms in sows infected with PPV virus depending on the virulence of strains and the time of gestation at which infection occurred [6]. The PPV virus can replicate and be shed from infected sows with no clinical symptoms, but transplacental infections usually result in death and mummification of the fetus before 70 days of gestation, in which fetuses have not developed antibodies to eliminate viruses and survived the infection. Constant infections of PPV in the herd and the inherent high mutation rate gave impetus to the emerging of new mutated strains [7, 8]. Vaccination against porcine parvovirus can’t prevent the virus infection and shedding, but it protects swine from SMEDI diseases [6].

PPV is a negative, single-stranded DNA virus with a genome about 5000 nucleotides, and its mature viron is an icosahedral symmetric particle with approximately 25 nm in diameter. The genome of PPV contains two large non-overlapping Open Reading Frames (ORFs), the 5′-end ORF, encoding a non-structural protein, and the 3′-end ORF, encoding three structural proteins VP1, VP2 and VP3 [9]. VP1 and VP2 were translated from alternatively spliced mRNA of the 3’-end ORF, while VP3 is a proteolytic product from VP2 by cleaving its 20 N-terminal residues in endosomes [10–12]. PPV capsids consist of 60 equivalent copies of heterotrimer that comprised of three structural proteins, VP1, VP2 and VP3 [13]. The VP2 protein is closely related to the virus-host range and antigenicity, and is generally considered as the major immunoprotective antigen of PPV vaccines, since it contains most of the B-cell epitopes critical to elicit neutralizing antibodies [14, 15].

VP2 proteins can spontaneously self-assemble into virus-like particles (VLPs), mimicking the morphology of pathogenic virus and maintaining an identical hemagglutination activity [16]. Recombinant PPV VLPs, first produced by the baculovirus expression vector system (BEVS), elicited high immune response that was identical to a commercial inactivated-virus vaccine [16]. BEVS is one of the most used systems for eukaryotic protein expressions, particularly for proteins that required posttranslational modifications or eukaryotic cellular milieus for proper folding [17]. However, high production costs constrained the extended use of BEVS in production of veterinary vaccines. Except for the BEVS, PPV VP2 has also been expressed in *Saccharomyces cerevisiae*, *Pichia pastoris* and *Escherichia coli*. In *S*. *cerevisiae* recombinant VP2 spontaneously assmebled into VLPs intracellularly, but only 8–9 mg VLPs were recovered from one liter of induced yeast culture [18]. *Pichia pastoris*, another yeast that is widely used for protein expression, produced the 595.76 mg/L VP2 proteins via secretory expression, but whether VLPs were formed extracellularly remains unknown [19]. *E. coli* is the most popular bacterial systems of producing recombinant protein, while most of VP2 proteins formed inclusion body after induction even if it was coexpressed with chaperones GroES-GroEL-tig [20].

PPV VLPs are safe and practical alternatives to inactivated infectious viruses as a vaccine that can provoke a strong protective immune response without the risk of diseases and offer a ready platform for facilitating recognition, uptake, and processing by the immune system [21]. As self-assembling proteinaceous nanoparticles, they were also used to deliver epitopes from other viruses. PPV VLPs were modified to display the CD8+ T-cell epitope from the lymphocytic choriomeningitis virus (LCMV) nucleoprotein induced strong cytotoxic T lymphocyte (CTL) responses and completely protected mice against a lethal LCMV infection [15, 22]. Similarly, mice vaccinated with PPV VLPs carrying B and T cell epitopes from foot-and-mouth disease virus (FMDV) VP1 protein elicited high neutralizing antibody titers against FMDV [23]. However, when PPV VP2 was fused with porcine circovirus 2 (PCV2) immunoreactive epitopes, residues 165–200 of Cap protein, at its N-terminus, hybrid PPV VLPs formed in HEK-293 cells induced specific antibodies response to both PPV and PCV2 in vaccinated mice [24].

In the current study, we sought to achieve a high production of PPV VLPs using a nonconventional yeast *Kluyveromyces marxianus*. This strain is an aerobic, Crabtree negative, and homothallic hemiascomycetous yeast that can generate energy from both respiratory metabolism and ethanol fermentation [25–27]. It has been developed for several biotechnological applications, such as the production of enzymes, heterologous proteins, bioingredients, and anticholesterolemic agents, and a host for synthetic biology platform, mainly due to its thermotolerance, high growth rate, broader substrate spectrum and generally recognized as safe (GRAS) [28–31]. Using *K. marxianus*, we achieved a 2.5 g/L VLPs of the Kresse strain in a 5 L fermentor. This production is much higher than previous reports. PPV VLPs produced in *K. marxianus* elicited high titers of IgG antibody and hemagglutination inhibition antibody, and therefore can be used to develop anti-PPV veterinary vaccines.

## Materials and methods

### Strains and plasmids

Laboratory strain *K*. *marxianus* Fim-1Δ*URA3* is a uracil auxotroph strain originated from *K. marxianus* Fim-1 deposited in China General Microbiological Culture Collection Center (CGMCC No.10,621 [25]. The vector pUKDN115 was constructed from the pUKD-S-PIT plasmid by replacing the fragment containing an α factor signal peptide sequence and human interferon α-2a gene with a multiple cloning site (MCS) [32].

### Construction of the recombinant strain

According to the *K. marxianus* codon preference, the *VP2* gene of Kresse strain (GenBank U44978.1) was optimized and synthesized by Genewiz Biotechnology Co., Ltd (Suzhou, China). The optimized *VP2* gene was deposited in the NCBI GenBank database under an accession number MT932328. The synthetic *VP2* gene was amplified with Phanta® Super Fidelity DNA Polymerase (Vazyme, Nanjing, China) using the following oligonucleotide primers (underlining indicates the homologous sequences from pUKD-N115 vector): 5′-TTTTTTTGTT AGATCCGCGG ATGAGCGAAA ACGTGGAGC-3′) and 5′-AGCTTGCGGC CTTAACTAGT CTAGTACAAC TTTCTTGGG-3′. The amplicon was ligated with the *Eco*R I and *Hin*d III linearized pUKDN115 by Gibson assembly [33], and then directly transformed into the FIM-1 Δ*URA3* strain according to the Lithium acetate transformation method [34]. Transformants formed on the SD plates (0.67% YNB, 2% glucose, 2% agar) were verified by PCR using the primers ATGAGCGAAA ACGTGGAGC-3 and CTAGTACAAC TTTCTTGGG-3′, and the positive clone was designated to the KM-PPV-VP2 strain.

### Expression and identification of the VP2 Protein in ***K. marxianus***

Fresh clones of KM-PPV-VP2 were inoculated in 50 mL YD medium (2% yeast extract, 4% glucose) and cultured at 30 °C, 220 rpm for 72 h. One milliliter of yeast cells harvested by centrifugation was washed with 1 ml PBS buffer (137 mM NaCl, 2.7 mM KCl, 10 mM Na_2_HPO_4_, 1.8 mM KH_2_PO_4_, pH 7.4) twice, and then suspended in 500 µl lysate buffer (50 mM HEPS, 140 mM NaCl, 1 mM EDTA, 1% Triton X-100, 0.1% Na-Deoxycholate, pH 7.5). Approximately 400 µl of glass beads (G8772, Sigma-Aldrich, Missouri, USA) was added to disrupt cells on a Bead-beater (FastPrep-24, MP, California, USA) at 6 m/s for 2 min. Cell lysates were centrifuged at 12,000 rpm, 4 °C for 20 min, and supernatants were used for SDS-PAGE and Western blotting assays. An anti-PPV VP2 polyclonal antibody and a goat anti-mouse IgG alkaline phosphatase-conjugate (074-1806, KPL, USA) was used as the primary and secondary antibody in western blotting respectively.

### Preparation of the anti-PPV VP2 polyclonal antibody

The native VP2 gene of Kresse strain was cloned into a pET-28a (+) vector within the *Sac* I and *Not* I sites ( (Novagen, Madison, USA), generating the pET-28a/VP2 plasmid. After transformation into *E. coli* BL21(DE3), VP2 protein was induced by 0.2 mM isopropyl-β-d-thiogalactopyranoside (IPTG), and purified by Ni-NTA (Ni Smart Beads, Smart-lifesciences, Changzhou, China) affinity basically as previously described [35]. Six weeks old Balb/C mice, purchased from Beijing Vital River Laboratory Animal Technology Co., Ltd, were immunized with 20 µg of purified VP2 antigen mixed with an equal volume of Freund’s adjuvant. After 35 days post-immunization (dpi), sera were separated and used as a primary antibody for Western blotting described above.

### Transmission Electronic Microscopy (TEM)

TEM scans of PPV VLPs were performed on a JEM-2100 Electron Microscope (JEOL Tokyo, Japan) according to Bucarey et al. [36]. Briefly, samples were spotted onto carbon-coated copper grids. After adsorption at room temperature for 5 min, copper grids were dried with filters and negatively stained with 3% of phosphotungstic acid (PTA). The grids were examined at an accelerating voltage of 120 kV.

### High cell‐density fermentation

High cell-density fermentation was conducted in a 5 L fermentor (BXBIO, Shanghai, China) as described recently [25]. The KM-PPV-VP2 strain was inoculated in 200 mL YD medium, grown at 30 °C, 220 rpm for 18 h, and then transferred into a fermentor containing 2 L defined mineral medium [25]. During fermentation, the dissolved oxygen was maintained above 10%, and the temperature was controlled at 30 °C. The pH was controlled automatically at 5.5 with ammonium hydroxide. At given intervals, 10 ml of culture was harvested to determine the cell density (OD_600_ nm) and wet cell weight (WCW). For SDS-PAGE analysis of VP2 productions, cell samples were diluted 1:10 with PBS buffer before disruption using the glass bead disruption described above. VLPs quantification was performed on an Agilent Series 1100 System (Agilent, Waldbronn, Germany) using a TSKgel G4000 SWXL column (300 mm × 7.8 mm i.d.) (Tosoh Bioscience, Stuttgart, Germany) and a TSKgel SWXL guard column (40.0 mm × 6.0 mm i.d.) (Tosoh Bioscience) as previously described [37].

### Screen of the ion‐exchange chromatography (IEX) media

KM-PPV-VP2 cells were collected by centrifugation at 5000 rpm for 10 min, followed by washing with deionized water twice. Rinsed cells were then suspended with PBS buffer pH7.4 for cation exchange, or with 20 mM Tris-HCl buffer pH 8.0 for anion resins. Cell lysates were prepared by high-pressure homogenization on a JN-02C Homogenizer (JNBIO, Guangzhou, China) under a condition of 1500 bar, 4 °C for 2 times, followed by centrifugation at 10,000 rpm, 4 °C for 30 min.Screens of IEX media were performed on the Poly-Prep®Chromatography Columns (Bio-Rad, Hercules, CA, USA) packaged 2 ml of different cation or anion resins listed in Table 1 as described previously [38]. Note that, in case of cation resins, pHs of cell lysates disrupted with PBS buffer should be adjusted to pH 4.0 with acetic acid before centrifugation. After washing with 5 volumes of 20 mM acetate buffer pH 4.0/Tris-HCl buffer pH 8.0, bound proteins were eluted by 5 mL of PBS containing 1M NaCl, and elutions were analyzed by SDS-PAGE.

**Table 1:** Ion exchange resins used in this work

Properties	Resins	Binding conditions	Manufacturers
Cation	Capto S ImpAct	20 mM NaAc-HAc pH4.0	GE healthcare
	Nuvia S		Bio-Rad
	SP Bestarose FF		Bestchrom
	Capto MMC		GE healthcare
	SP HP		Bestchrom
	CM Sepharose FF		GE healthcare
	POROS HS		Life Science
	CaptoSP ImpRes		GE healthcare
	Nuvia cPrime		Bio-Rad
Anion	Capto Q XP	20 mM Tris-HCl pH8.0	GE healthcare
	Capto Q		GE healthcare
	Q Bestarose FF		Bestchrom

### Purification of the PPV VLPs

To purify PPV VLPs by IEX chromatography, yeast cells were suspended in PBS buffer pH7.4 and disrupted by high-pressure homogenization. Cell lysates were subsequently adjusted to pH 4.0. After centrifugation at 10,000 rpm, 4 °C for 30 min, the supernatants of pH adjusted cell lysate were loaded onto an XK 50/30 column (GE Healthcare) packed with 400 mL of Capto S ImpAct resin. Binding VLPs were eluted with 20 mM sodium acetate buffer containing 500 mM NaCl. To elevate the recovery of VLPs, the precipitates of pH adjusted cell lysates were redissolved in an equal volume of 20 mM Tris-HCl buffer pH 8.0. Through centrifugation, the clarified supernatant were loaded onto an XK 50/30 column packed with 400 mL of Capto Q XP resins. After elution with 20 mM Tris-HCl buffer pH 8.0 plus 500 mM NaCl, fractions were diafiltrated for 10 volumes of PBS on ÄKTA flux (GE Healthcare, USA) equipped with a 750 kDa column (11-0005-50, GE healthcare). Further polishing purification of PPV VLPs was performed on an AKTA Purifier 100 (GE Healthcare, USA) using a HiPrep™ 26/60 Sephacryl® S-500 HR column (GE Healthcare). About 4 ml IEX purified sample was injected and eluted with PBS at a rate of 0.5 mL/min. Protein concentration was measured by the BCA Protein Assay Kit (23,250, Thermo Fisher Scientific).

### Vaccination of mice with PPV VLPs

Purified VLPs were diluted with PBS buffer and emulsified with MONTANIDE™ Gel 01 adjuvant (Seppic, Paris, France) at a rate of 10%, giving a final antigen concentration of 240 µg/mL. Fifteen of 6-week old female SPF Balb/c mice were randomly divided into 3 groups (n = 5). Grouped mice were subcutaneously injected with 20 µg, 40 µg PPV VLPs, and 250 µL of PBS as a control, respectively. Blood samples were collected from cheek each week till 49 dpi. Sera were separated by incubating blood at 37 °C for 1 h, followed by centrifugation at 3000 rpm for 4 min, and stored in small aliquots at − 20 °C.

### Antibodies detection by enzyme‐linked immunosorbent assay (ELISA)

96-well Costar Assay Plates (Corning, NewYork, USA) were coated with the Ni-NTA purified VP2 protein according to the method described previously [36]. ELISA detections of the anti-PPV IgG titers in mouse sera were performed as described previously by Duan et al. [38].

### Serum hemagglutination inhibition (HI) antibody assay

HI antibody titers of serum samples were determined in U-bottom 96-well plates according to the standard method [39]. Briefly, serum samples were inactivated at 56 °C for 30 min. Before the test, non-specific inhibitors of hemagglutinin in serum samples were removed by treating with 25% kaolin and 3% porcine erythrocytes. The treated sera were serially diluted 1:4 with PBS buffer, and each 25 µL of serum diluent was mixed with an equal volume of 40 µg/mL purified PPV VLPs. The plates were incubated for 1 h at 37 °C, and then 50 µL of 1% porcine erythrocyte was added per well. Finally, the plates were incubated at room temperature for 40 min to calculate the HI titers based on the reciprocal of the highest dilution inhibited hemagglutinin completely.

### Spleen lymphocyte proliferation and cytokine detection assay

At 42 dpi, three mice from the groups injected with 20 µg PPV VLPs and PBS were euthanized to separate spleen lymphocytes in a mouse lymphocyte separation medium (Dakewe, Beijing, China). The separated spleen cells were then cultivated in 96-well plates containing 100 µL of 1640 culture medium (Thermo Fisher Scientific, Illinois, USA), at a concentration of 3 × 10^6^ cells/mL. After adding 0.2 µg Concanavalin A (Sigma, MO, USA), the plates were incubated at 37 °C for 48 h. Proliferative responses were detected using the Cell Titer 96® AQueous One Solution Cell Proliferation Assay Kit (Promega, Madison, WI, USA), and stimulation indexes (SI) were calculated as the ratio of the stimulated sample divided to the unstimulated control at OD_490 nm_. Cytokines secreted by the spleen cells were measured using the Cytometric Bead Array (CBA) Mouse Th1/Th2 Cytokine Kit (Becton Dickinson Biosciences, San Jose, CA, USA).

## Results

### Expression of PPV VP2 in *K*. *marxianus*

**Fig. 1:**
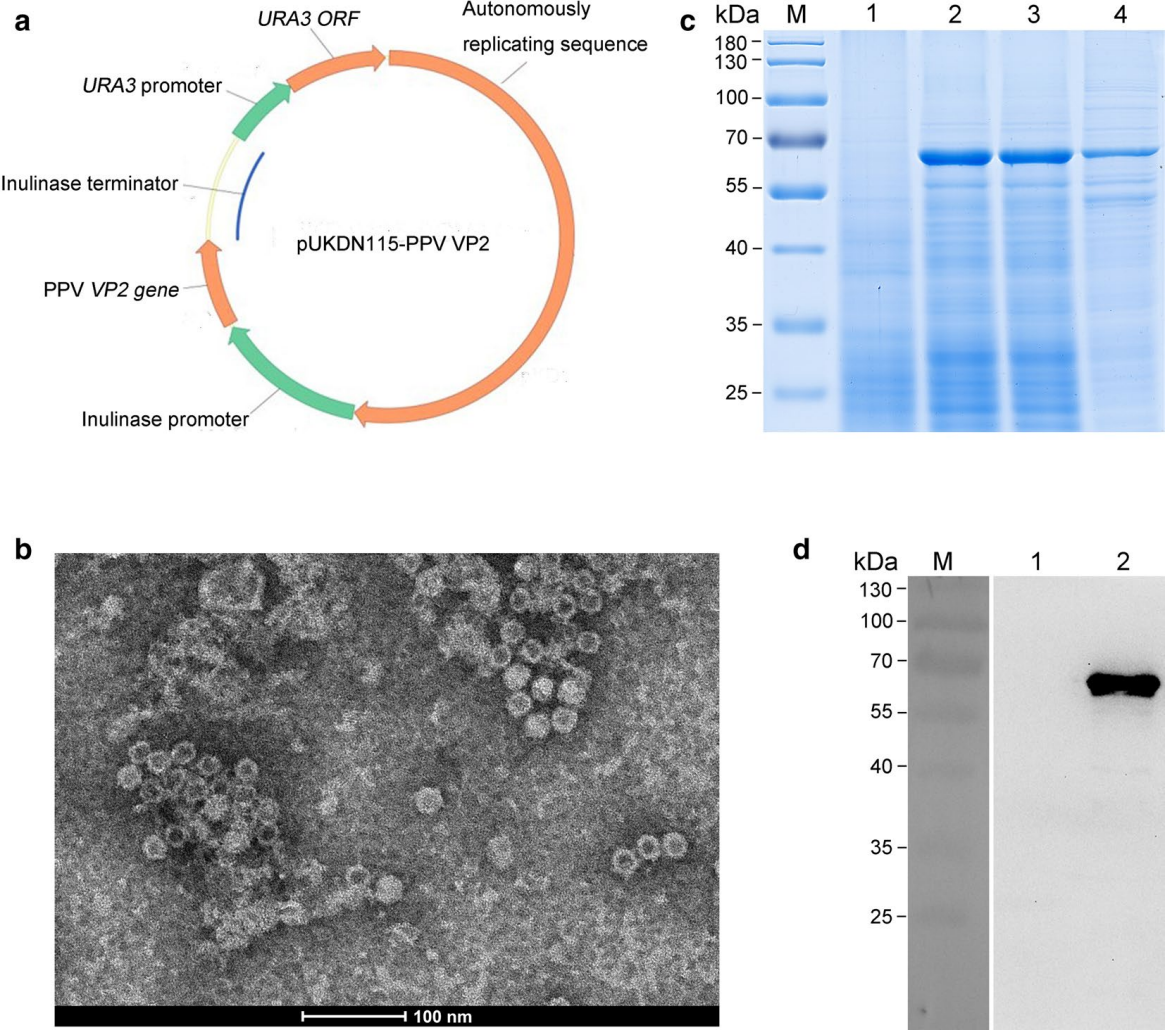
Expression and characterization of the PPV VP2 protein in *K*. *marxianus*. **a** Illustration of the recombinant expression plasmid pUKDN115-PPV VP2. The inulinase promoter, inulinase terminator, and URA3 cassette in pUKDN115 are all derived from *K*. *marxianus*, and autonomously replicating sequence is from pKD1. **b** TEM scan of the assembled PPV VLPs in *K*. *marxianus* intracellularly. SDS-PAGE (**c**) and Western Blot (**b**) assays of the lysates of recombinant strain after shaked for 66 h. Lane M: PageRuler Prestained Protein Ladder;Lane 1: the lysate of Fim-1 ∆*ura3* transformed with the empty plasmid pUKDN115; Lane 2: the lysate of KM-PPV-VP2; Lane 3: the supernatant of KM-PPV-VP2 cell lysate; Lane 4: the precipitate of KM-PPV-VP2 cell lysate

The *VP2* gene of the Kresse strain was used to express in *K*. *marxianus*, since this PPV strain displayed an increased virulence compared with other virulent strains that could kill immunocompetent fetuses [40]. The native VP2 coding sequence was redesigned for expression according to the codon usage bias of *K. marxianus*, and then inserted directly downstream of the *K. marxianus* inulinase promoter of the pUKDN115 vector (Fig. 1a). The resulting vector pUKDN115-VP2 was transformed into *K. marxianus* Fim-1 *ura3*∆ strain, generating a recombinant strain KM-PPV-VP2. For detection of the expression of VP2 protein, the KM-PPV-VP2 strain was cultured in YD medium for 72 h and cell lysates were subjected to SDS-PAGE and Western blotting respectively. Compared with Fim-1 *ura3*∆ transformed with the empty pUKDN115 vector, the KM-PPV-VP2 cell lysates contained an additional 65 kDa band, which was consistent with the theoretical molecular weight of VP2 protein (Fig. 1c). This VP2 protein band was further confirmed by Western Blotting with anti-PPV VP2 polyclonal antibody (Fig. 1d).

Previous studies revealed that the recombinant VP2 expressed in insect, eukaryotic or prokaryotic cells formed virus-like particles (VLPs) [16, 18, 19, 41, 42]. To test whether VP2 proteins were spontaneously assembled into VLPs in *K. marxianus*, cell lysates of KM-PPV-VP2 was scanned by TEM, and results confirmed that VP2 proteins were assembled into VLPs with a diameter of approximately 20 nm in *K. marxianus* (Fig. 1b).

### High production of PPV VLPs in fed‐batch fermentation

**Fig. 2:**
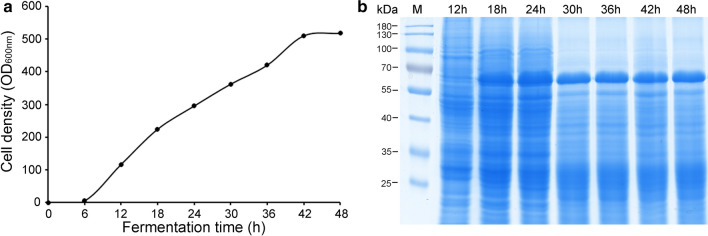
High cell-density fermentation of the KM-PPV-VP2 strain. **a** The growth curve of KM-PPV-VP2 in a 5L fermentor. **b** SDS-PAGE of the VP2 protein expression in KM-PPV-VP2 cell lysates at the indicated time. Yeast cells were collected at an interval of 6 h, and cells cultures were then diluted with PBS at a rate of 1:10. After disruption by high-pressure homogenization, each of 15 µl cell lysates was loaded to analyze by SDS-PAGE analysis

Scale-up production of VP2 protein was conducted in a 5 L reactor using a defined chemical medium. Throughout the fermentation, to achieve a high cell density of the KM-PPV-VP2 strain, glucose was fed at a limited rate to maintain the dissolved oxygen above 10%. After 48 hour fermentation, the cell density (OD_600 nm_) reached 560, with a dry biomass more than 120 g/L (Fig. 2a). At certain time points, KM-PPV-VP2 cells were disrupted to detect the expression of VP2 protein by SDS-PAGE. As shown in Fig. 2b, the VP2 began to accumulate intracellularly at 18 h, and at 48 hour the VP2 yield, quantified via SE-HPLC using the purified VLPs as a standard, was approximately 2.5 g/L.

### Screening IEX media to capture PPV VLPs from cell lysates

**Fig. 3:**
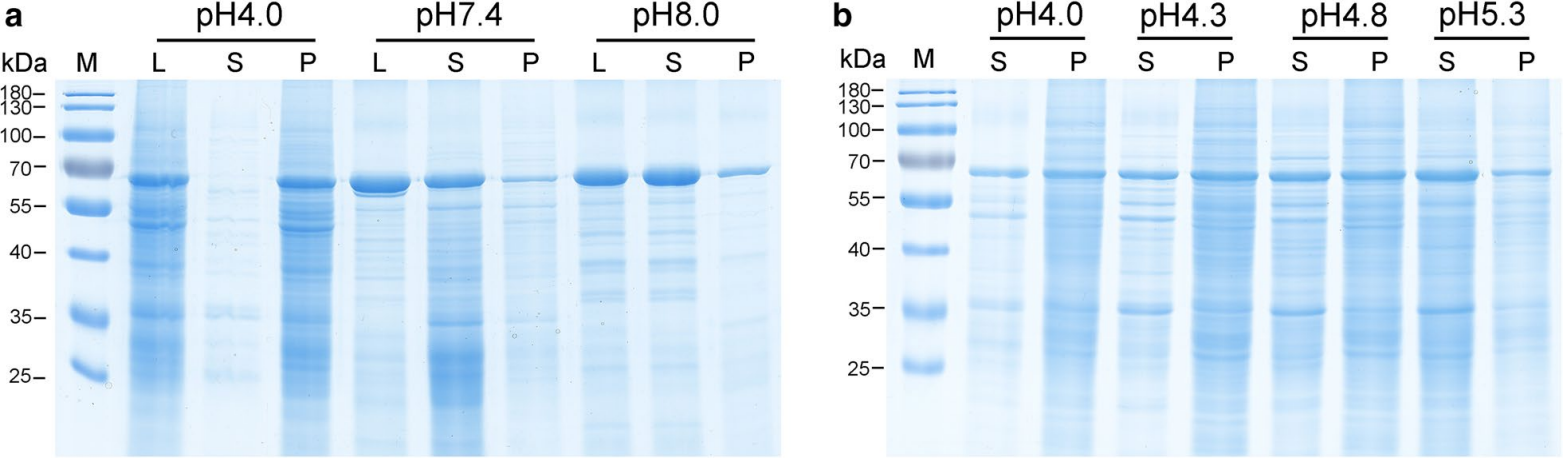
Effects of pH values on the solubility of PPV VLPs. **a** Detections of PPV VLPs released during cell disruption under the given pH values. **b** The solubility of PPV VLPs in pH adjusted cell lysates. KM-PPV-VP2 yeast cells were disrupted with PBS buffer pH7.4 and then adjusted by acetic acid to pH values ranging from 5.3 to 4.0. Lane L: cell lysates; Lane S: supernatants of cell lysates; Lane P: precipitates of cell lysates

To purify PPV VLPs by ion-exchange chromatography, pH effects on its solubility was investigated before screening of IEX media. Three buffers ranging from pH 4.0 to 8.0 were used to analyze the solublility of PPV VLPs when disrupting KM-PPV-VP2 cells by high-pressure homogenization. As shown in Fig. 3a, almost PPV VLPs existed in the precipitate when using 20 mM acetate buffer pH4.0, while they became soluble in cases of PBS buffer pH7.4 or 20 mM Tris-HCl buffer pH8.0 (Fig. 3a). Therefore, to obtain soluble PPV VLPs and to facilitate downstream purification steps, PBS buffer pH 7.4 was chose to disrupt the KM-PPV cells. Subsequently, KM-PPV cell lysates disrupted in PBS buffer pH 7.4 were adjusted to pH 5.3, 4.8, 4.3, and 4.0 with 100 mM acetic acid respectively. SDS-PAGE of these pH adjusted samples indicated that lowering pH decreased the solubility of PPV VLPs significantly, with approximately 50% remained in the precipitate at pH 4.0 (Fig. 3b).

**Fig. 4:**
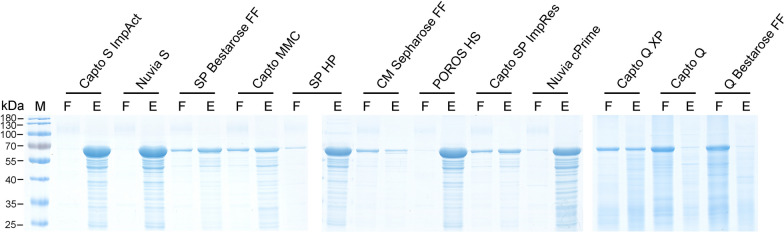
Screening the IEX chromatography media for purification of PPV VLPs. Cation exchange resins screen was performed using the KM-PPV-VP2 cell lysates that were disrupted with PBS buffer pH7.4 PBS and acidified to pH 4.0 with acetic acid. For anion exchange resins, samples were prepared by using 20 mM Tris-HCl as the buffer to suspend and disrupt the KM-PPV-VP2 cells. Lane F: flowthrough fractions; Lane E: elution fractions

Besides pH values, 12 cation or anion resins were used to investigated the potentials of them in the purification of PPV VLPs (Table 1). Experimental results were shown in Fig. 4. Among the used cation resins, the Capto S, Nuvia S, and POROS HS resins showed best performances to capture PPV VLPs, with few in the flowthrough fractions, but the SP HP, Nuvia cPrime, SP Bestarose FF, Capto MMC, Capto SP ImpRes, and Capto Q XP resins shared relatively weak affinities. However, when using cation CMFF resins or two anion resins, Capto Q and Q Bestarose FF, most of PPV VLPs were observed in flowthrough fractions, surggesting that these three resins were unsuitable for the IEX purification. Thus, we choose the Capto S ImpAct resin for cation IEX media due to its high binding capacity and good selectivity for PPV VLPs.

**Fig. 5:**
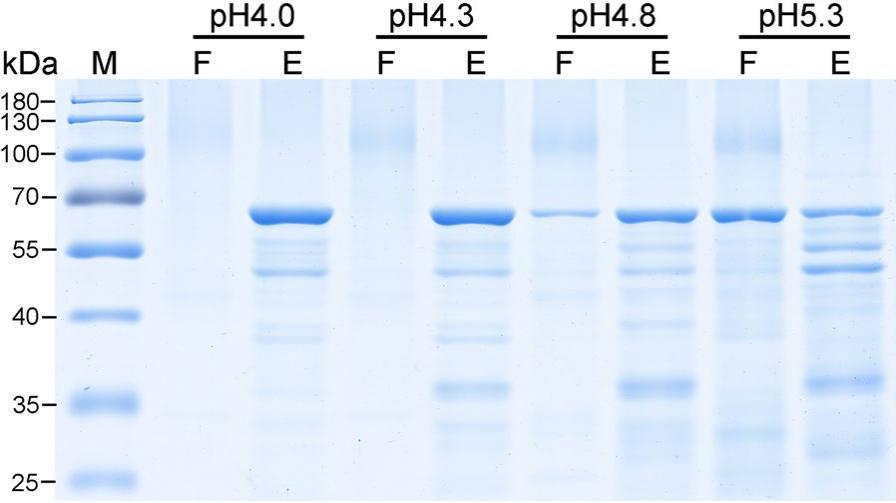
pH value effects on the cation exchange purification of PPV VLPs with Capto S ImpAct resin. The KM-PPV-VP2 cell lysates disrupted with PBS buffer pH7.4, and adjusted to pH 5.3–4.0 with acetic acid. After centrifugation at 12,000 rpm for 15 min, 2 ml supernatants were loaded onto Biorad Poly-Prep chromatography columns packaged with 1 mL Capto S ImpAct resins and eluted PBS pH7.4 containing 0.5 M NaCl. Lane F: flow-through fractions; Lane E: elution fractions

Subsequently, the supernatants with different pHs described above were used to determine the optimal pH for Capto S ImpAct resins in binding of PPV VLPs. These data were presented in Fig. 5. Overall, PPV VLPs were bound to Capto S ImpAct resins at a pH range from 4.0 to 5.3, but the impurities at pH 4.0 were relatively less than that of other pHs.

### Purification of PPV VLPs

**Fig. 6:**
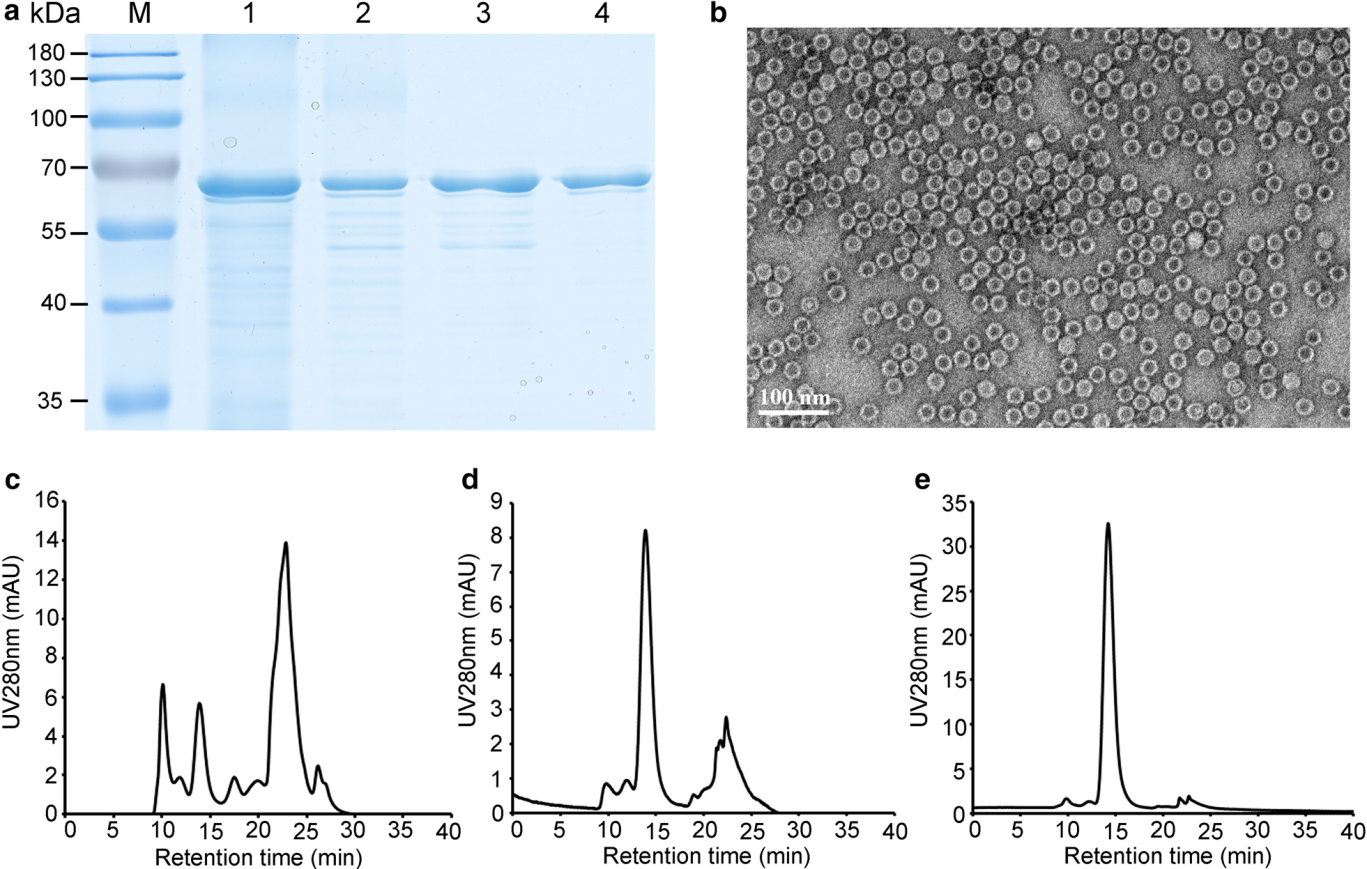
Scale-up purification of PPV VLPs. **a** SDS-PAGE analysis of the purified PPV VLPs samples from IEX and Gel filtration. Lane M, PageRuler Prestained Protein Ladder; Lane 1, cell lysates prepared by PBS pH 7.4; lane 2, the supernatant of cell lysates adjusted to pH 4.0; Lane 3, elution fraction of Capto S ImpAct exchange chromatography; Lane 4, elution of Sephacryl® S-500 Gel filtration. **b** TEM scan of the Gel filtration purified PPV VLPs. Bar, 100 nm. c HPLC analysises of the supernatant pH adjusted cell lysate (**c**), the IEX purified PPV VLPs (**d**), and the gel filtration purified PPV VLPs (**e**)

Based on the pHs and resin screens, two steps, the IEX chromatography and Sephacryl® S-500 gel filtration, were set out to purify the PPV VLPs expressed by *K. marxianus*. To facilitate the cation exchange purification of PPV VLPs, the KM-PPV-VP2 cell lysates were adjusted to pH 4.0 and supernatants was separated for the IEX purification by Capto S ImpAct resins. As a result, about 250 mL elution fraction containing 2.73 mg/mL PPV VLPs was obtained from 1 L fed-batch fermentation cultures (Fig. 6a). The purity of PPV VLPs in the IEX elution fraction reached about 64%, which was detemined by the HPLC method. Thereafter, elution fraction was subjected to a polishing step, Sephacryl® S-500 gel filtration, to remove trace impurities. After two-step purification, the purity of PPV VLPs reached above 95%, with a recovery of 30%. To confirm the conformation of the purified PPV VLPs, samples collected from the polishing purification step were examined by TEM (Fig. 6b), and results showed that the purified VLPs remained their original shapes.

**Fig. 7:**
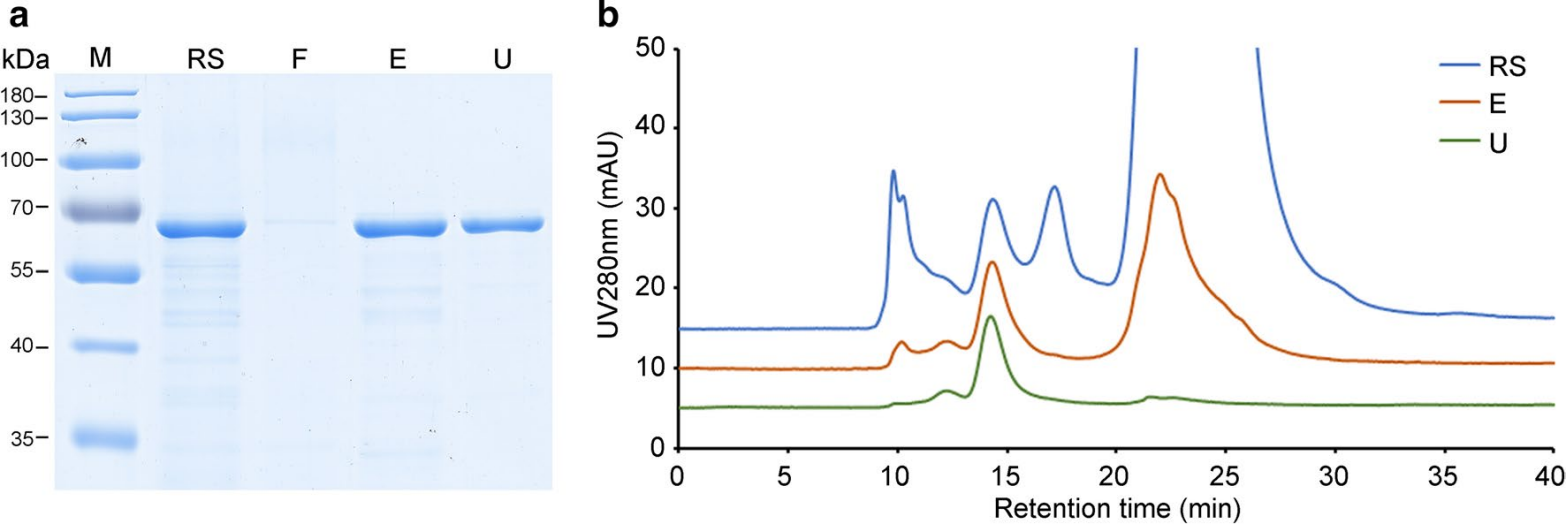
SDS-PAGE (**a**) and HPLC (**b**) assays of the purified PPV VLPs remained in the pH adjusted precipitates. The precipitates were dissolved using 20 mM Tris-HCl buffer pH 8.0. After clarification by centrifugation, supernatants were loaded onto a Capto Q XP column and PPV VLPs were eluted with 20 mM Tris-HCl pH 7.4 containing 0.5 M NaCl. Elution fractions were ultrafiltrated with a 750 kDa column in a tangential flow filtration. Lane RS: the redissolved supernatant; Lane F: flowthrough fractions; Lane E: elution fractions; Lane U; the ultrafiltrated VLPs

However, the pH adjustment KM-PPV-VP2 cell lysates resulted that approximately half of PPV VLPs were remained in the precipitates. To recover this part of antigen, we introduced an anion exchange purification process using Capto Q XP resin. The precipitates were suspended with an equal volume of 20 mM Tris-HCl buffer pH8.0 to the cell lysates to dissolve the precipitated PPV VLPs. After repeat centrifugation, the supernatants were loaded onto an XK 50/30 column packaged Capto Q XP resins. From SDS-PAGE, we found PPV VLPs were almost captured by the anion resins, and the purity of elution was lower than 10% as analyzed by HPLC (Fig. 7b). HPLC analysis also revealed the main impurities of anion-exchange chromatography were small molecules, whose molecular weights were far smaller than PPV VLPs. Thus, to remove these impurities, the elution fractions were diafiltrated by a crow-flow mode with a 750 kDa column. After that the purity of PPV VLPs reached higher than 95% (Fig. 7b). Generally, by combination of anion and cation exchange chromatography, the total recovery of PPV VLPs from KM-PPV-VP2 cell lysates increased to above 60%.

### Antibody response in the mice

**Fig. 8:**
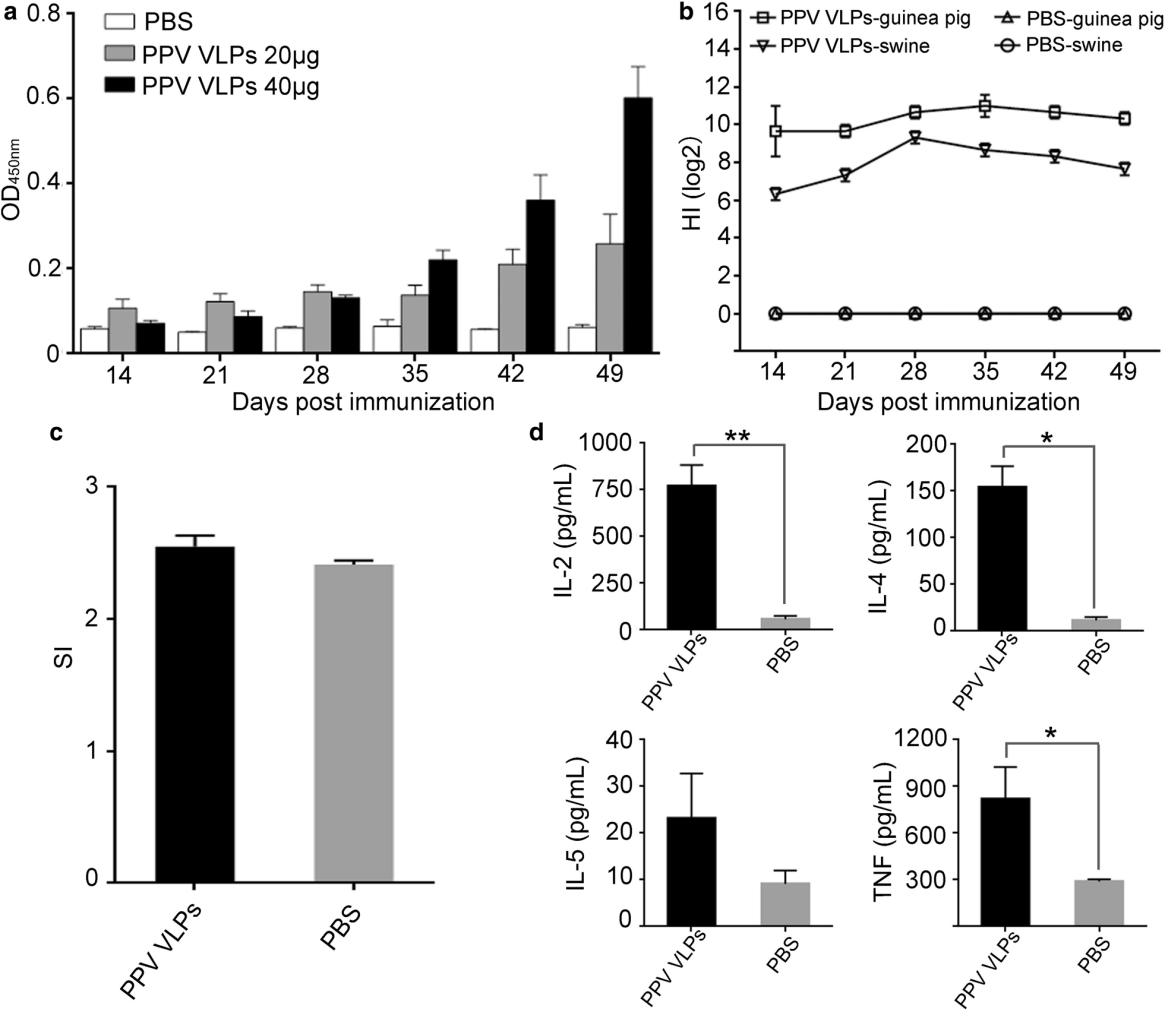
Immunogenicity assays of the PPV VLPs vaccine. a The titers of anti-PPV IgG in mouse sera immunized with PPV VLPs. Mice in each group (n = 5) were immunized with 20 µg or 40 µg of the PPV VLPs vaccine, and PBS as a control. b the HI antibody titers of mouse immunized with 20 µg PPV VLPs. Bars represented mean ± SEM (n = 5). c The stimulation indexes of mouse lymphocytes. At 56 dpi, spleen lymphocytes of the immunized mice and PBS group were isolated and then were stimulated in triplicate with conA. d The levels of cytokines secreted by the mice lymphocytes at 56 dpi. * P < 0.05; ** P < 0.01

To investigate the immunogenicity of recombinant PPV VLPs prepared by *K. marxianus*, different doses of purified PPV VLPs antigen that were emulsified with MONTANIDE™ Gel 01 adjuvant were subcutaneously injected into SPF Balb/c mice. After 14 dpi, mice sera were separated to measure PPV specific antibodies by ELISA assay every 7 days. As shown in Fig. 8a, PPV-specific antibodies were detected in vaccinated mice sera after 14 dpi. In both immunized groups, antibody levels increased with the days post-immunization. However, the titers of PPV-specific antibody in mice immunized 40 µg antigen were significantly higher than those of 20 µg, indicating that mice immunized with 40 µg of PPV VLPs could effectively produce high-level IgG antibodies.

In the hemagglutination assays, the PPV VLPs derived from Kresse strain showed strong hemagglutination activity on both porcine and guinea pig erythrocytes. Assays for hemagglutination inhibition activities of immunized mice sera were then conducted with 40 µg/mL purified VLPs and serially diluted porcine/guinea pig erythrocytes. After just immunized for 14 dpi, the 2log titers of HI reached around 6 for the PPV specific antibodies, while the mice sera injected with PBS could not inhibit the aggregation of PPV VLPs on pig blood cells (Fig. 8b). After 28 dpi, HI titers in nearly all mice rose to values about 8.

### Spleen lymphocyte proliferation and cytokine detection

The spleen lymphocytes isolated from mice after 42 dpi showed promptly response to conA stimulation in vitro proliferation and consequently giving a higher SI than the negative control group (Fig. 8c). This result indicated that PPV VLPs acted as strong immunogen to provoke the cellular immune responses. At the same time, to investigate the induction of humoral immunity spleen lymphocytes we opted to detect the levels of cytokines IL-2, IL-4, IL-5 and TNF–α in spleen lymphocytes cultures after stimulation for 72 h. On average, all the tested cytokines, including IL-2, IL-4, IL-5 and TNF, were higher in immunized spleen lymphocyte cultures than those of PBS groups (Fig. 8d). These results suggested that PPV VLPs produced by *K*. *marxianus* could induce good humoral immunity as well.

## Discussion

The commercially available PPV vaccines are inactivated whole-virus and live-attenuated vaccines. Irrespective of safety risks such as incomplete inactivation and reversion to virulence, albeit small probability, there are intrinsic disadvantages involved with the administration of inactivated or attenuated vaccines that it hampered to discriminate the differentiation of infected from vaccinated animals (DIVA) in seropositive animals [43]. Intrigued by the inherently safe vaccine and useful epitope carrier nanoparticles [15], we developed *K. marxianus* as a new platform for the production of VLPs. In previous study, we efficiently produced virus-like particles of porcine circovirus (PCV) type 2 by expressing the capsid Cap protein in *K*. *marxianus * [38]. However, PCV is one of the smallest animal viruses and its Cap protein is only 28 kDa in size. To investigate the potential and versatility of *K*. *marxianus* to express large capsid protein, assembly of large capsids, and production of different VLPs, the PPV VP2, may be among the largest capsid protein of known viruses, was used to express in our *K*. *marxianus* strain. Attribute to high growth rate and facile high biomass, the recombinant *K. marxianus* strain produced 2.5 g/L PPV VLPs after just a 48 h fermentation. As a comparison, the production level of VLPs in *Pichia pastoris*, *E. coli*, *S. cerevisiae* is 595.76 mg/L [19], 15 mg/L [35], and 8–9 mg/L [18] respectively. Moreover, *K. marxianus* has a notable advantage over other strains, such as *S. cerevisiae* and *E. coli*, on high production of VLPs that there is no additional inducement during its fermentation since glucose serves as the carbon resources to support cell growth as well as an inducer for its inulinase promoter to obtain high expression of heterologous proteins [25].

As to yeast cells, there is no additional treatment to remove endotoxins, but other downstream operations, such as disrupting the rigid wall of the yeast cells to release VLPs, clarification of cell lysates, and purification processes, are the main factors that significantly increased the production costs overall [44]. In previous studies, the PPV VLPs were commonly purified by sucrose or cesium chloride gradient centrifugation [18, 45], or Ni-NTA affinity chromatography [20, 41], which are not conducive to large-scale production. Aiming to create a simple and low-cost purification process, we assessed the ion-exchange chromatography for purification of PPV VLPs comprehensively. Due to the low isoelectric point of VP2 protein, yeast cell lysates should be acidified to pH 4.0, in order to cationize PPV VLPs and facilitate the anion resins binding. Simultaneously, this treatment could reduce the turbidity of cell lysates apparently, with most of the impurities settled. However, about 50% of VLPs were precipitated during this process. As an alternative strategy to elevate the recovery, we dissolved the precipitated VLPs by 20 mM Tris-HCl buffer pH 8.0, in which PPV VLPs are highly soluble, and PPV VLPs were then purified by anion-exchange chromatography coupled with a cross-flow diafiltration. Owing to the first round of charification, most of negatively charged competitors including nucleic acid, polysaccharides, and small molecules in yeast cell lysates were removed, which could decrease the efficiency of Capto Q XP resins in binding of PPV VLPs (Figs. 4 and 7). The purified PPV VLPs from the precipitates gave a purity of 95%, which was comparable to the gel-filtration purification. By this means, less than 40% of PPV VLPs were lost after these purification processes. These findings provides a new guideline for PPV VLPs purification from intracellular yeast cells.

Currently, most of inactivated or attenuated PPV vaccines were originated from the Cluster A strain NADL-2. The Kresse strain, isolated in the USA in 1985, is the most serious type of PPV-1 that can kill the immunocompetent fetuses [46]. These two strains have a high identity both in sequence and genomic organization, but have quite different pathogenicities [11]. Compared with the NADL-2 strain, three substitutions in VP1/VP2 protein structurally located on the surface of its capsid that may contribute to the highly virulent of PPV Kresse strain [47]. A recent study revealed the genotypes of porcine parvoviruses isolated in South Korea in 2018 was more identical to the ancient Kresse strain. This re-evolution to its original strains may increase the epidemiologic risk of PPV [48]. Additionally, the spread of PPV-27a, a unique virulent cluster D strain that has a unique immunological feature, raised the concerns about the efficacy of currently used inactivated or attenuated vaccines. A recent report revealed that a “Kresse-like” K22 PPV strain-based vaccine showed stronger protection than the commercial NADL-2 based vaccines in a PPV-27a strain challenge [49]. Enlightened by these, we set out to express the VP2 capsid protein of Kresse strain and produce PPV VLP antigens in our *K. marxianus* expression platform. Another, recombinant VLPs can be easily changed to desired genotype based on a well-established expression platform in a shorter time.

Compared to inactivated PPV vaccines, facile high titers of PPV VLPs elicited better cellular immune responses in gilts, and induced high neutralization antibody and high hemagglutination inhibition antibody in guinea pigs [20, 50]. When vaccinated with doses of 20–40 µg per mouse PPV VLPs from *K. marxianus*, high titers of specific IgG antibodies were induced in mice. As is known, PPV virus could agglutinate erythrocytes of chicken, guinea pig, mouse, human, monkey, rat, and cat [51]. In our study, sera of mice immunized with Kresse strain VLPs showed high hemagglutination inhibition, a serologic index to the specific antibody of a virus, against either swine or guinea pig erythrocytes, surggested that VLPs produced in *K. marxianus* maintained the natural morphology of pathogenic virus [52]. But the cause of hemagglutination to swine erythrocytes by the Kresse strain VLPs is unknown. Except for the antibodies, Spleen lymphocyte proliferations and cytokines detection further confirmed that this vaccine has a good immune effect, caused both humoral and cellular immunity. The commercial whole-virus vaccines, based on the nonpathogenic NADL2 strain, protected pigs against PPV disease but do not prevent infection and virus shedding when challenged with an antigenically heterologous strain PPV-27a [6, 51, 53]. Furthermore, an experimental vaccine based on the PPV-27a strain protected the fetuses from vaccinated sows against disease when infected with the homologous PPV strain [54]. These results suggest the PPV VLPs produced in this study can be used to protect swine from porcine parvovirus diseases.

## Abbreviations

PPV: Porcine parvovirus
SMEDI: Stillbirth, mummification, embryonic death, and infertility
ORFs: Open Reading Frames
VLPs: Virus-like particles
MCS: Multiple cloning sites
IPTG: Isopropyl-β-d-thiogalactopyranoside
dpi: Days post-immunization
PTA: Phosphotungstic acid
IEX: Ion exchange chromatography
DIVA: Differentiation of infected from vaccinated animals
